# OX40 Expression in Eosinophils Aggravates OVA-Induced Eosinophilic Gastroenteritis

**DOI:** 10.3389/fimmu.2022.841141

**Published:** 2022-06-02

**Authors:** Longwei Xu, Dan Tian, Minsi Zhou, Jiuyue Ma, Guangyong Sun, Hua Jin, Mingyang Li, Dong Zhang, Jing Wu

**Affiliations:** ^1^ Department of Gastroenterology, Peking University Ninth School of Clinical Medicine, Beijing, China; ^2^ Beijing Key Laboratory of Tolerance Induction and Organ Protection in Transplantation, Beijing, China; ^3^ Immunology Research Center for Oral and Systemic Health, Beijing Friendship Hospital, Capital Medical University, Beijing, China; ^4^ Beijing Clinical Research Institute, Beijing, China; ^5^ Department of Gastroenterology, Beijing Friendship Hospital, Capital Medical University, Beijing, China; ^6^ National Clinical Research Center for Digestive Diseases, Beijing, China

**Keywords:** eosinophils, OX40, type 2 immunity, NF-κB signaling pathway, eosinophilic gastrointestinal diseases

## Abstract

**Background & Aims:**

Eosinophils are the main inflammatory effector cells that damage gastrointestinal tissue in eosinophilic gastrointestinal diseases (EGIDs). Activation of the OX40 pathway aggravates allergic diseases, such as asthma, but it is not clear whether OX40 is expressed in eosinophils to regulate inflammation in EGIDs. In this study, we assessed the expression and effect of OX40 on eosinophils in WT and *Ox40^-/-^* eosinophilic gastroenteritis (EGE) mice.

**Methods:**

Eosinophil infiltration, ovalbumin (OVA)-specific Ig production, OX40 expression and inflammatory factor levels in the intestine and bone marrow (BM) were investigated to evaluate inflammation.

**Results:**

We confirmed that OVA-challenged mice produced high levels of *Ox40, Mbp, Ccl11, Il5, Il4, Il13, and Il6* mRNA and a low level of *Ifng* mRNA in the intestine. Increased eosinophils were observed in intestinal and lymph tissues, accompanied by significantly upregulated OX40 and Type 2 cytokine production in eosinophils of EGE mice. *Ox40* deficiency ameliorated OVA-induced inflammation, eosinophil infiltration, and cytokine production in the intestine. Consistently, *Ox40^-/^* ^-^ eosinophils exhibited decreased proliferation and proinflammatory function. The stimulation of the agonistic anti-OX40 antibody, OX86, promoted the effect of OX40 on eosinophils. The present study also showed that *Ox40* deficiency dampened the Traf2/6-related NF-κB signaling pathway in eosinophils.

**Conclusions:**

OX40 may play a critical role in the progress of OVA-induced EGE by promoting the maturation and function of eosinophils *via* the Traf2/6-related NF-κB signaling pathway.

## Introduction

The end-effector cells of eosinophilic gastrointestinal diseases (EGIDs), eosinophils, are associated with gastrointestinal tissue injury. Several studies observed that the tissue sites of EGIDs were infiltrated by degranulated eosinophils (1–3) and immune mediators. Antigen-presenting cells in the mucosal layer and the lamina propria layer (LPL) of the gastrointestinal tract recognize food antigen molecules and present them to Th0 cells, which differentiate into IL-5 and IL-13-secreting Th2 cells (4). IL-5, secreted by Th2 cells, promotes the differentiation, proliferation, and maturation of bone marrow (BM) eosinophils (5, 6). Mature eosinophils undergo chemotaxis to the inflammatory site in intestinal tissue driven by Th2 cell-derived eotaxin-1, eotaxin-2, and eotaxin-3. By releasing numerous intracellular granules that include major basic protein (MBP), eosinophil peroxidase (EPX), eosinophil cationic protein (ECP), and eosinophil-derived neurotoxin (EDN), eosinophils cause serious tissue damage (2, 7, 8). In addition, eosinophils also express a variety of other immune mediators, such as IL-1, IL-3, IL-4, IL-5, IL-13, transforming growth factor β, tumor necrosis factor, and eotaxin, that further damage tissues and aggravate the inflammatory reaction (2, 9, 10).

OX40 (CD134), which is expressed by T cells, macrophages (11) and neutrophils (12), participates in adaptive immunity and innate immunity (13) by promoting proliferation, survival, differentiation, and even cytokine production and accelerating inflammation (14, 15). OX40 promotes the occurrence and development of allergic diseases, such as asthma (16). OVA-challenged *Ox40^-/-^* mice were shown to exhibit less lung eosinophil infiltration; lower levels of IL-4, IL-5, and IL-13; a weaker Th2 immune response; and less severe lung inflammation (16). Previous studies have focused mainly on T cells, however, whether OX40 is expressed by eosinophils is still unknown. As previously described, eosinophils are the main effector cells in EGIDs and promote intestinal inflammation by releasing MBP, EPX and pro-inflammatory cytokines (6, 17). However, it is not clear whether OX40 promotes the development of allergic diseases by regulating eosinophils.

The OVA-induced eosinophilic gastroenteritis (EGE) animal model is widely used to explore the immunologic mechanism involved in the pathogenesis of EGE. Here, we showed that the *Ox40* mRNA level was elevated in the intestinal tissue of OVA-challenged mice. In addition, OVA challenge significantly upregulated OX40 expression in eosinophils. *Ox40* deficiency ameliorated OVA challenge-induced inflammation, increased eosinophil apoptosis and reduced eosinophil proliferation. Furthermore, *Ox40* deficiency limited the production of type 2 inflammatory factors by eosinophils both *in vivo* and *in vitro* by dampening the TNFR-associated factors (Trafs) 2/6-related NF-κB signaling pathway.

## Methods

### Mice

Eight- to ten-week-old male and female C57BL/6J WT control mice was purchased from Beijing HFK Bioscience (Beijing, China), and *Ox40* ^–/–^ mice on the C57BL/6 background was purchased from the Jackson Laboratory (Bar Harbor, Maine, USA). Mice were kept under specific pathogen–free (SPF) conditions at Peking University Ninth School of Clinical Medicine and Beijing Friendship Hospital. All procedures were performed in accordance with the guidelines of the Institutional Animal Care and Ethics Committee at Peking University Ninth School of Clinical Medicine and Beijing Friendship Hospital. The IACUC number was 21-2009.

### Mouse Model of OVA-Induced Eosinophilic Gastroenteritis

**Figure 1:**
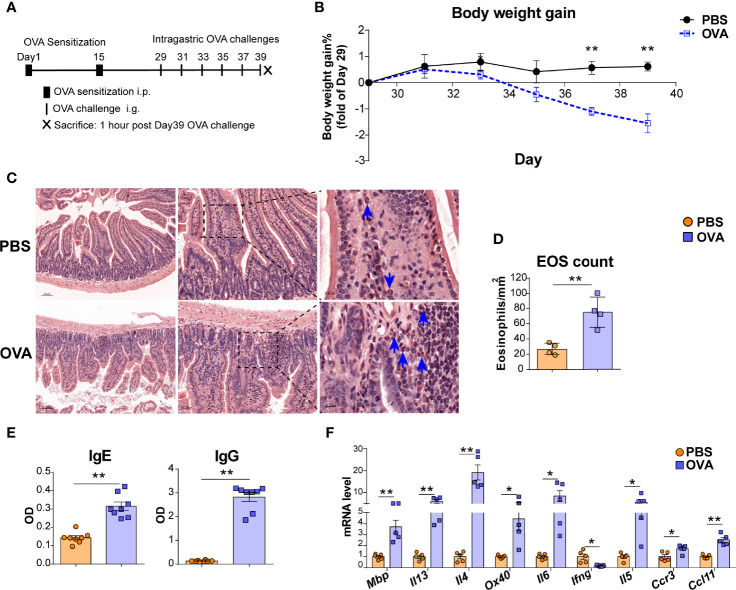
OVA challenge upregulated intestinal eosinophils infiltration and OX40 expression in intestine tissue. **(A)** Schematic of the EGE mouse model. IP, intraperitoneal. IG, intragastric. **(B)** Body weight gain of the two groups (n=4-5 mice for each group). **(C)** Small intestine sections were stained with Hematoxylin Congo Red staining to measure eosinophils infiltration and calculate the count of eosinophils. **(D)** The statistical analysis of the counts of eosinophils in Hematoxylin Congo Red staining sections. **(E)** Optical density (OD) of plasma OVA-specific IgE and IgG levels of control and OVA-treated WT mice detected by ELISA. **(F)** Relative mRNA levels of *Mbp, Il13, Il4, Ox40, Il6, Ifng, Il5, Ccr3*, and *Ccl11* in intestinal tissue (n=4-8 mice for each group). Experiments were repeated 2-3 times. Data are represented as the mean ± SEM. *P < 0.05; **P < 0.01.

EGE was induced as previously described (18). In brief, eight- to ten-week-old female C57BL/6J WT mice and *Ox40-KO* (*Ox40* ^–/–^) mice were systemically sensitized with 50 µg ovalbumin (OVA) (A5503, Sigma-Aldrich, MO, USA) and 1 mg aluminum potassium sulfate dodecahydrate (A6435, Sigma-Aldrich) dissolved in a 0.5 ml 0.9% sodium chloride solution on Day 1 and Day 15. Beginning from Day 29, mice were intragastrically challenged with 50 mg OVA dissolved in 0.25 ml phosphate-buffered saline (PBS) every two days for a total of 6 times. Age- and sex-matched C57BL/6J and *Ox40^–/–^* mice were used as controls and were systemically sensitized with 0.9% sodium chloride and intragastrically challenged with PBS. Mice were euthanized, and the blood, BM, spleen, mesenteric lymph nodes (mLNs), small intestine and colon were harvested 1 hour after challenge on Day 39 (**Figure 1A**).

### Body Weight Gain

As diarrhea was hard to quantitate in mice, the body weight gain was calculated to assess the severity of diarrhea. The body weight was measured every two weeks before intragastric stimulation and every two days after gavage stimulation. And body weight gain was calculated as percentage of weight gain from Day 29.

### Peripheral Blood Processing

Mouse peripheral blood was collected with an EDTA anticoagulation tube and centrifuged for isolation of peripheral blood cells and plasma. Plasma was frozen immediately at -80°C and subsequently used to detect OVA-specific IgG and IgE antibodies by ELISA. After an incubation with flow cytometry antibodies, red blood cells (RBCs) in peripheral blood cells were lysed with Lysing Solution (349202, BD FACS™), and the remaining cells were washed with PBS for quantitative analysis of eosinophils by flow cytometry.

### Intestinal Tissue Processing

Small intestine and colon tissue samples were prepared as previously described (19). In brief, the small intestine and colon were separated carefully, and all mesenteric fat was discarded. The gut lumen was rinsed by flushing with PBS, and Peyer’s patches (PPs) were lifted from the surface of the small intestine. Then, the small intestine and colon were opened longitudinally and cut into pieces for H&E and Hematoxylin-Congo red staining, RNA extraction, and enzymatic digestion into single cells for flow cytometric analysis.

### HE and Hematoxylin-Congo Red Staining

Eosinophils in the small intestine were identified by Hematoxylin-Congo red staining as previously described (20). Briefly, sections were stained in Hematoxylin for 5 minutes at room temperature and followed by rinsing in running tap water. Then, 0.5% alcoholic Congo red solution (C6277, Sigma-Aldrich) was added for staining for 15 minutes at room temperature. Finally, 75% alcohol solution was used to differentiate the sections for a few seconds. Eosinophils were calculated by counting the orange-red cytoplasmic cells in sections of the small intestine with the assistance of PANNORAMIC Digital Slide Scanners and CaseViewer (3DHistech, Budapest, HUN) and expressed as eosinophils/mm^2^. Four to five section areas were randomly selected per animal for eosinophil assessment. Tissue sections were also prepared for H&E staining to observe the tissue structure and inflammation.

### Isolation of Single Cells From the Spleen, mLNs, Small Intestine LPL and Colon LPL

The spleen and mLNs were mechanically disrupted to generate single-cell suspensions and passed through a 70-µm filter (352350, BD Biosciences, Falcon, USA) as previously described (21). To clarify whether the treatment of collagenase and erythrocyte lysis buffer affects the detection of cell surface proteins, the surface markers of splenic cell were assessed by flow cytometry after the treatment of collagenase D (11088866001, Sigma (Roche), USA) and erythrocyte lysis buffer (79217, QIAGEN, Germany). As shown in **Figure S1**, collagenase D and lysis buffer treatment did not damp the detection of surface antigens by flow cytometry. The small intestine and colon were cut into several 1- to 2-cm pieces. The epithelial cells were removed by incubating the pieces of intestine in a pre-digestion solution for 20 min at 37°C with rotation (120–130 rpm/min). The pre-digestion solution was 1X HBSS containing 10 mM EDTA (EDS-100G, Sigma) and 1 mM DL-dithiothreitol solution (DTT) (43816-10 ML, Sigma). After pre-digestion, the tissue pieces were digested with collagenase D and DNase I (DN25-5G, Sigma) and mechanically ground with a gentleMACS™ Octo Dissociator (Miltenyi Biotec GmbH) according to the manufacturer’s protocol. Cells derived from intestine and colon tissues were purified with 30% Percoll (10303540, Cytiva, USA) and centrifugation for 20 min at 500xg at room temperature. Finally, the cell pellet was resuspended and washed with ice-cold PBS.

### Generation of Bone Marrow-Derived Eosinophils and Purification of Mature BM Eosinophils

Male WT and *Ox40^-/-^* mice were used to obtain bone marrow eosinophils. After treatment with erythrocyte lysis buffer to remove RBCs, bone marrow cells were used for culture and sorting of mature BM eosinophils. Unselected mouse bone marrow progenitors harvested from WT or *Ox40* ^–/–^ mice were cultured with recombinant mouse (rm)FLT3-L (100 ng/ml, 250-31L, PeproTech, USA) and rmSCF (100 ng/ml, 250-03, PeproTech) for 4 days, followed by culture with rmIL-5 (10 ng/ml, 215-15, PeproTech) alone thereafter for 4-6 days to generate bone marrow-derived eosinophils (bmEos) as previously described (22). On Days 8-10, cultured eosinophils were purified with a FACS Aria II cell sorter (BD Biosciences, CA, USA) using gating on 7AAD-Siglec-F+SSC-A^hi^ cells as previously described (22). For natural BM eosinophils, mature BM eosinophils were sorted as previously described (23). Briefly, B cells (anti-B220), T cells (anti-CD3), DCs (anti-CD11c), neutrophils and macrophages (anti-Gr-1) were depleted by MACS. Then, the remaining cells were incubated with anti-7AAD, anti-FcϵRIα, anti-Siglec-F, and anti-CD11b antibodies. Live mature eosinophils, gated as 7AAD-FcϵRIα-Siglec-F+CD11bint cells, were sorted with a FACS Aria II cell sorter.

### Flow Cytometric Analysis

Single cells from the blood, spleen, mLNs, bone marrow, small intestine LPL and colon LPL were obtained as described above. The cells were stained with mouse Fc-blocking reagents for 5-10 minutes at 4°C, followed by an incubation with conjugated surface marker-specific antibodies for 30 minutes at 4°C. Then, the cells were washed with MACS buffer (0.5% bovine serum albumin in PBS) twice, finally resuspended in MACS buffer, and analyzed by flow cytometry on an Attune NxT (Invitrogen, Thermo Fisher Scientific) and a FACS Aria II flow cytometer (BD Biosciences). As for intracellular antigens detection, cells were treated with the cell activation cocktail (423304, Biolegend) for 4 hours before surface staining. After surface staining, cells were fixed and Permeabilized by Cyto-Fast™ Fix/Perm Buffer Set (426803, Biolegend) in the dark for 20 minutes at room temperature. Fixed/permeabilized cells were resuspended in Intracellular Staining Perm Wash Buffer and the fluorophore-conjugated antibodies or an appropriate negative control were added and incubated for 20 minutes in the dark at room temperature. Finally, cells were washed twice before detection. FlowJo software (Tree Star, OR, USA) was used to analyze the data. Live and dead cells were distinguished with 7-AAD and Zombie Aqua™ Dye. The antibodies used were listed in **Table 1**.

**Table 1:** Antibodies used for flow cytometry in this study.

Manufacturer	Mouse Antibody	Clone/Catalog number
Biolegend (San Diego, CA, USA)	CD45 (PE/Cyanine7)	S18009F
	CD45 (FITC)	QA17A26
	CD45 (BV711)	30-F11
	B220 (PE)	RA3-6B2
	CD11c (PE)	N418
	CD11c (PE/ Cyanine7)	N418
	CD11c (PE/ Cyanine5)	N418
	CD3 (PE)	17A2
	Gr-1 (PE)	RB6-8C5
	FceRIa (PE/Cyanine7)	MAR-1
	Siglec-F (APC)	S17007L
	Siglec-F (FITC)	S17007L
	Siglec-F (PE)	S17007L
	Annexin V (PE)	640934
	Annexin V (PE/Cyanine7)	640949
	7-AAD (Percp/Cyanine5.5)	420404
	Ly6G (APC/Cyanine7)	1A8
	Ly6G (Pacific Blue)	1A8
	CD11b (BV421)	M1/70
	CD11b (BV605)	M1/70
	Ki67 (Alexa Fluor 647)	16A8
	Ki67 (PE)	16A8
	BCL-2 (FITC)	BCL/10C4
	IFNγ (BV711)	XMG1.2
	IFNγ (BV605)	XMG1.2
	IFNγ(PE/Cyanine7)	XMG1.2
	IL-4 (PE)	11B11
	IL-4 (APC)	11B11
	IL-13 (PE)	W17010B
	TNF-α (PE/Cyanine7)	MP6-XT22
	IL-10 (PE/Cyanine7)	JES5-16E3
	IL-6 (PE)	MP5-20F3
	CD16/32	156604
	OX40 (APC)	OX-86
	OX40 (PE)	OX-86
	MHCII (Percp/Cyanine5.5)	M5/114.15.2
	MHCII (BV785)	M5/114.15.2
	CCR3 (PE)	J073E5
	CCR3 (FITC)	J073E5
	Zombie AquaTM Dye	423102
Abcam(Cambridge, MA, USA)	NFκB P105/P50	Ab32360
	Goat pAb to Rb IgG (Alexa Fluor 488)	Ab150077
Cell Signaling Technology (MA, USA)	pNF-κB p65 (S536) (PE)	5733s

### Purification of Intestinal Eosinophils

The small intestine single cell suspension of WT and *Ox40^-/-^* mice was obtained as described above, and incubated with anti-CD45, anti-CD11b, anti-Siglec-F, and anti-Ly6G, anti-F4/80, anti-CD11c, anti-MHCII, anti-CCR3, and anti-7AAD antibodies in the dark for 30 minutes at 4 °C. Finally, cells were washed twice. After being excluded by CD11b+Ly6G+ (neutrophils), CD11b+F4/80+ (macrophages) and CD11c+MHCII+ (DCs), the eosinophils were identified as CCR3+Siglec-F+ cells and sorted by a FACS Aria II cell sorter.

### Ox86 Stimulation and rm IL-5 Stimulation of Sorted Eosinophils

Sorted intestinal eosinophils of C57BL/6J WT and *Ox40* ^-/-^ mice were treated with 10-30 ng/ml IL-5 for 1-6 hours for 7AAD, Annexin V, Ki67, and mRNA levels of cytokine detection. BM eosinophils and intestinal eosinophils sorted from C56BL/6J WT mice were stimulated with OX86 (BioXCell, West Lebanon, NH, USA) or Rat-IgG for 6-12 hours. Stimulated cells were harvested for flow cytometric and RT–PCR analysis.

### RT–PCR

Total RNA was extracted from small intestine tissue, cultured bmEos and sorted mature BM eosinophils and intestinal eosinophils with TRI reagent (T9424-200ML, Sigma) and reverse transcribed into cDNA with the PrimeScript RT Reagent Kit (RR037A, TaKaRa, Japan) according to the manufacturer’s protocol. q-PCR was performed using an ABI 7500 Sequence Detection System (Applied Biosystems, CA, USA). Relative gene expression was quantified using the 2-ΔΔCt method. The primer sequences of the target genes are shown in **Table 2**.

**Table 2:** Primer sequences detected by real-time PCR.

Gene	Strand	Primer sequence (5'-3')
*GAPDH*	Forward	AAGGTCATCCCAGAGCTGAA
	Reverse	CTGCTTCACCACCTTCTTGA
*Ox40*	Forward	GGGCAGGGAACACAGTCAAC
	Reverse	CAGAATTGCACACCTACTCAG
*Traf1*	Forward	GGAGGCATCCTTTGATGGTA
	Reverse	AGGGACAGGTGGGTCTTCTT
*Traf2*	Forward	GCCTTTCCAGATAACGCTGC
	Reverse	TCGTGGCAGCTCTCGTATTC
*Traf3*	Forward	GAACCTGCTGAAGGAGTGGA
	Reverse	GACTCGTTGTTTCGGAGCAT
*Traf4*	Forward	CCGGCTTCGACTACAAGTTC
	Reverse	TCAGGGCATTTGAAGACTCC
*Traf5*	Forward	CGCACCTGTCCCTGTACTT
	Reverse	AGGCAATGTTCATCTCGCCA
*Traf6*	Forward	ATCCATAAGGGATGCAGGGC
	Reverse	GGCACTTTACCGTCAGGGAA
*Epx*	Forward	TAGGGGCCTTAGCCACACTC
	Reverse	CTGCTATGCAGTCTCGAAGGA
*Mbp*	Forward	TCAGTGTTAACTTCGGAATCCA
	Reverse	TCTTGACACAGTGAGATAGACG
*Il4*	Forward	GTCATCCTGCTCTTCTTT
	Reverse	ATGGCGTCCCTTCTC
*Il13*	Forward	GCAGCATGGTATGGAGTGTG
	Reverse	TGGCGAAACAGTTGCTTTGT
*Il6*	Forward	GTTCTCTGGGAAATCGTGGA
	Reverse	GGAAATTGGGGTAGGAAGGA
*Il5*	Forward	GCAATGAGACGATGAGGCTTC
	Reverse	GCCCCTGAAAGATTTCTCCAATG
*Ifng*	Forward	GGCCATCAGCAACAACATAAGCGT
	Reverse	TGGGTTGTTGACCTCAAACTTGGC
*Ccl11*	Forward	GATCTTCTTACTGGTCATGATAAAGCA
	Reverse	TGTCTCCCTCCACCATGCA
*Ccr3*	Forward	TGCTGAGATGTCCCAATA
	Reverse	TCACCAACAAAGGCGTAG
*Il10*	Forward	AGAAAAGAGAGCTCCATCATGC
	Reverse	TTATTGTCTTCCCGGCTGTACT
*Il12p35*	Forward	CAATCACGCTACCTCCTCTTTT
	Reverse	CAGCAGTGCAGGAATAATGTTTC
*Tnfα*	Forward	TCCCAGGTTCTCTTCAAGGGA
	Reverse	GGTGAGGAGCACGTAGTCGG

### OVA-Specific IgE and IgG Analysis

OVA-specific IgG and IgE in plasma samples were detected as previously described (24). Briefly, high-binding plates were coated with 2 μg/ml OVA in carbonate buffer at 4°C overnight and blocked with 2% milk/PBS. Then, the plates were incubated with 1:5 serum dilutions for IgE detection and 1:100,000 serum dilutions for IgG detection. An HRP-conjugated rat anti-mouse IgG antibody (ZB -2305, Zsbio, Beijing, China) and anti-mouse IgE antibody (PA1-84764, ThermoFisher (Invitrogen), USA) were used to detect OVA-specific IgG and OVA-specific IgE, respectively. After an incubation with a standard TMB solution for 5-10 minutes, an ELISA stop solution was added, and the optical density (OD) was read at 450 nm on a microplate reader within 15 minutes.

### Statistics

Results were analyzed using Student’s t-test or nonparametric Mann–Whitney test among two groups and ANOVA among four groups by a statistical software package (GraphPad Prism). A *P* value of 0.05 or less was considered statistically significant.

## Results

### OVA Challenge Significantly Upregulated Ox40 Expression in Eosinophils From Intestine Tissue

An OVA challenge-induced EGE mouse model was used to explore whether OX40 is expressed in eosinophils (**Figure 1A**). As shown in **Figure 1B**, OVA challenge led to decreased body weight, obvious intestinal eosinophils infiltration (orange-red cytoplasmic cells with blue arrows in **Figures 1C, D**), inflammatory cells infiltration and destruction of intestinal villi and crypts (**Figure S2A**), lower levels of OVA specific IgG and IgE (**Figure 1E**) and upregulated inflammatory gene (*Mbp*, *Il4, Il13, Il6, Il5, Ccl11*, *Ccr3*, and *Ox40*) expression (**Figure 1F**).

**Figure 2:**
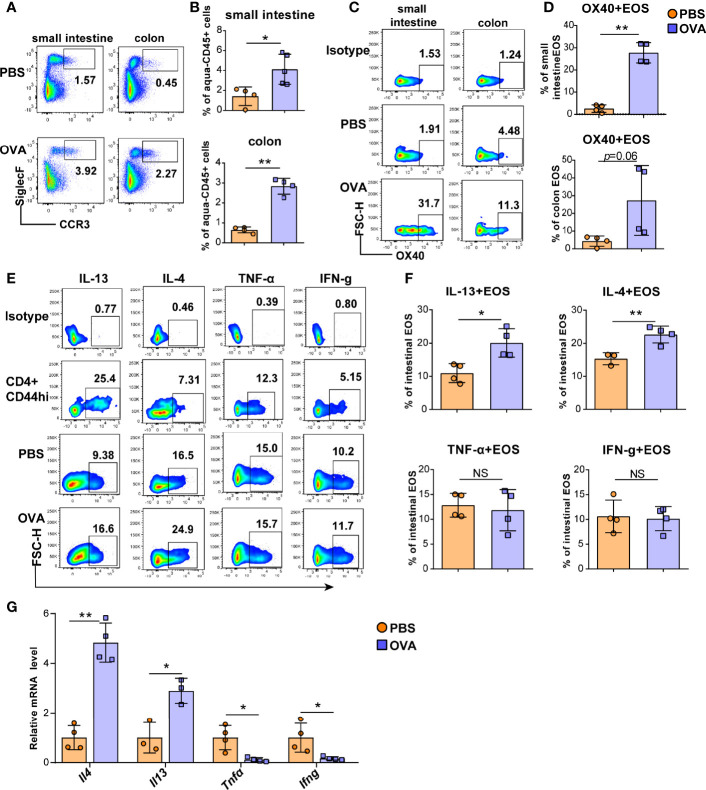
OVA challenge upregulated intestinal eosinophils infiltration and OX40 expression and Th2 cytokine production. Eosinophils in the small intestine LPL and colon LPL were detected by flow cytometry **(A)** and analyzed by statistical methods **(B)**. OX40 expression in eosinophils of the small intestine LPL and colon LPL was detected by flow cytometry **(C)** and analyzed by statistical methods **(D)**. The typical flow cytometry results **(E)** and statistical analysis **(F)** of IL13, IL-4, IFN-g, and TNF-α expression in eosinophils and CD4+CD44+ T cells of small intestine. **(G)** Intestinal eosinophils of OVA challenged and PBS treated mice were sorted with a FACS Aria II cell sorter to measure the mRNA levels of *Il13, Il4, Ifng*, and *Tnfα* (n=3-5 mice/group). Experiments were repeated 2-3 times. Data are represented as the mean ± SD. *P < 0.05; **P < 0.01; NS, nonsignificant.

Eosinophils cause intestinal inflammation in EGE *via* degranulation and type 2 cytokine production. To quantify eosinophils infiltration, eosinophils from the small intestine and colon LPLs, spleen, mesenteric lymph nodes (mLNs), bone marrow (BM) and peripheral blood were detected by flow cytometry, and the gating strategy was shown in **Figures S3A, B**. Elevated proportions of eosinophils were observed in the small intestine LPL, colon LPL (**Figures 2A, B**), mLNs, and peripheral blood (**Figures S4A, B**) in the OVA-induced EGE model. In particular, OVA challenge significantly upregulated OX40 expression in eosinophils in the small intestine LPL and there was an increasing trend in the colon LPL (**Figures 2C, D**). Similarly, sorted mature BM eosinophils stimulated with rmIL-5 for 12-24 hours exhibited up-regulated OX40 expression (**Figure S5**). Otherwise, the IL-4 and IL-13 expression in eosinophils were increased, while the IFN-g and TNF-α expression were similar in the two groups in small intestinal eosinophils (**Figures 2E, F**). Real-time PCR was used to detect the mRNA levels of cytokines of sorted intestinal eosinophils of mice in the two groups. As shown in **Figure 2G**, the mRNA levels of *Il4* and *Il13* were markedly increased and the mRNA levels of *Ifng* and *Tnfα* were significantly decreased in OVA challenged group. Besides, eosinophils in mLNs expressed a higher level of IL-13 (**Figures S6A, B**) in OVA challenged mice. These data suggest a possible critical role for OX40 in eosinophils in EGE mice.

### OVA Challenge Induced BM Eosinophils to Synthesize More Type 2 Cytokines and Express Higher Levels of Ox40

**Figure 3:**
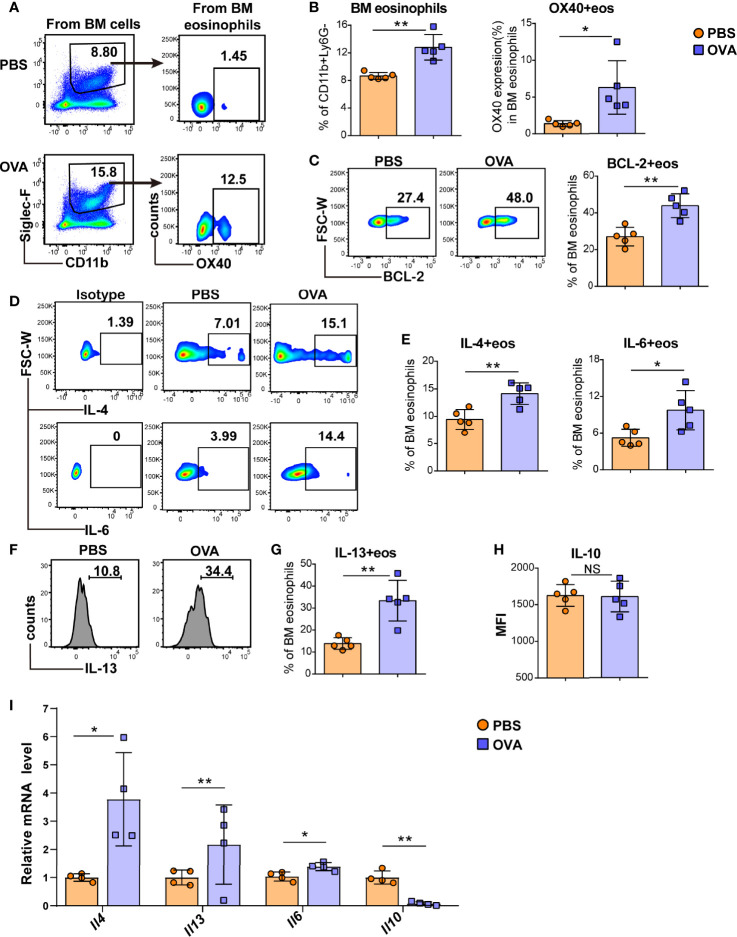
OVA challenge induced BM eosinophils to synthesize more type 2 cytokines. Typical flow cytometry results **(A)** and statistical analysis **(B)** of BM eosinophils and OX40 expression in BM eosinophils from PBS-treated and OVA-treated WT mice. BCL-2 expression in BM eosinophils was detected by flow cytometry and analyzed by statistical methods **(C)**. Representative flow cytometric analysis **(D, F)** and statistical analysis **(E, G, H)** of IL-4, IL-6, IL-13, and IL-10 expression in BM eosinophils in the two groups. **(I)** Relative mRNA levels of *Il13, Il4, Il6*, and *Il10* of sorted bone marrow eosinophils were detected in the two groups (n=4-5 mice/group). Experiments were repeated 2-3 times. Data are plotted as the mean ± SD. *P < 0.05; **P < 0.01; NS, nonsignificant.

As peripheral eosinophils are all derived from the bone marrow, we further investigated eosinophils in the BM of EGE mice. Consistent with the OX40 expression of intestinal eosinophils, OVA challenge induced more BM eosinophils and a higher level of OX40 in bone marrow eosinophils (**Figures 3A, B**). Furthermore, the expression of the anti-apoptotic protein BCL-2 (**Figure 3C**) was significantly elevated in OVA-challenged BM eosinophils. Compared to those of mice in the control group, the BM eosinophils of OVA-challenged mice secreted more IL-4, IL-6, and IL-13 (**Figures 3D–G**). However, no difference in IL-10 (**Figure 3H**) expression was observed between the two groups. At the mRNA level, sorted BM eosinophils in OVA challenged mice expressed higher levels of *Il4, Il13* and *Il6* mRNA and lower level of *Il10* mRNA (**Figure 3I**). Therefore, OVA challenge promoted the production of type 2 immunity-related cytokines by eosinophils in the BM.

### Ox40 Deficiency Ameliorates Intestinal Inflammation and Eosinophil Activation Induced by OVA Challenge

**Figure 4:**
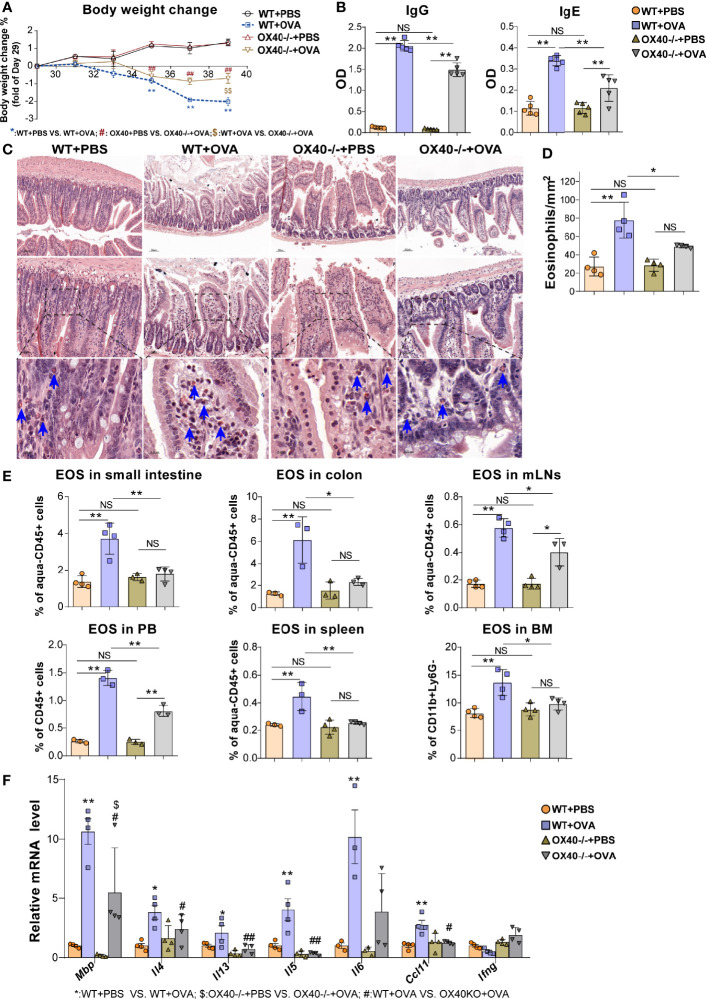
*Ox40* deficiency ameliorates OVA challenge-induced inflammation. The body weight change **(A)** and plasma OVA-specific IgE and IgG levels **(B)** of mice were detected in the four groups. Small intestine sections were stained with Hematoxylin Congo red staining **(C)** and statistical analysis of the counts of infiltrated eosinophils in Hematoxylin Congo red staining **(D)**. **(E)** Statistical analysis of the eosinophils proportion in the small intestine LPL, colon LPL, mLNs, spleen, peripheral blood, and bone marrow. **(F)** Relative mRNA levels of *Mbp, Il4, Il13, Il5, Ifng, Ccl11*, and *Il6* in intestinal tissue in each group (n=3-5 mice/group). Experiments were repeated 2-3 times. Data are represented as the mean ± SEM. *P < 0.05; **P < 0.01; ^#^P < 0.05; ^##^P < 0.05; ^$^P < 0.05; NS, nonsignificant.

To further verify the role of OX40 in OVA-induced EGE, WT mice and *Ox40* ^–/–^ mice were sensitized and challenged with OVA. Compared to WT mice, *Ox40* ^–/–^ mice showed higher body weight gain (**Figure 4A**), lower plasma OVA-specific IgG and IgE levels (**Figure 4B**). Eosinophils infiltration was detected by flow cytometry (gating strategy was shown in **Figure S3A**) and Hematoxylin-Congo red staining. Eosinophils were stained as orange-red cytoplasmic cells (with blue arrows in **Figure 4C**) in Hematoxylin-Congo red staining, and calculated eosinophils counts were analyzed by statistical methods. There were fewer infiltrating eosinophils in intestinal tissue (**Figures 4C, D**), and less destruction of villus and crypt structures (**Figure S2B**) in OVA challenged *Ox40* ^–/–^ mice. Similar results were verified by flow cytometry, there were fewer eosinophils infiltrating the small intestine LPL, colon LPL, spleen, mLNs, BM, and peripheral blood in *Ox40* ^–/–^ mice than in WT mice (**Figure 4E**) after OVA challenge. Compared to WT mice, *Ox40* ^–/–^ mice showed decreased mRNA levels of cytokines that promote the chemotaxis and activation of eosinophils, including *Mbp*, *Il13, Il5*, *Il4 and Ccl11* (**Figure 4F**) after OVA challenge.

### Ox40 Deficiency Inhibits the Survival and Cytokine Production of Intestinal Eosinophils *in Vivo*

**Figure 5:**
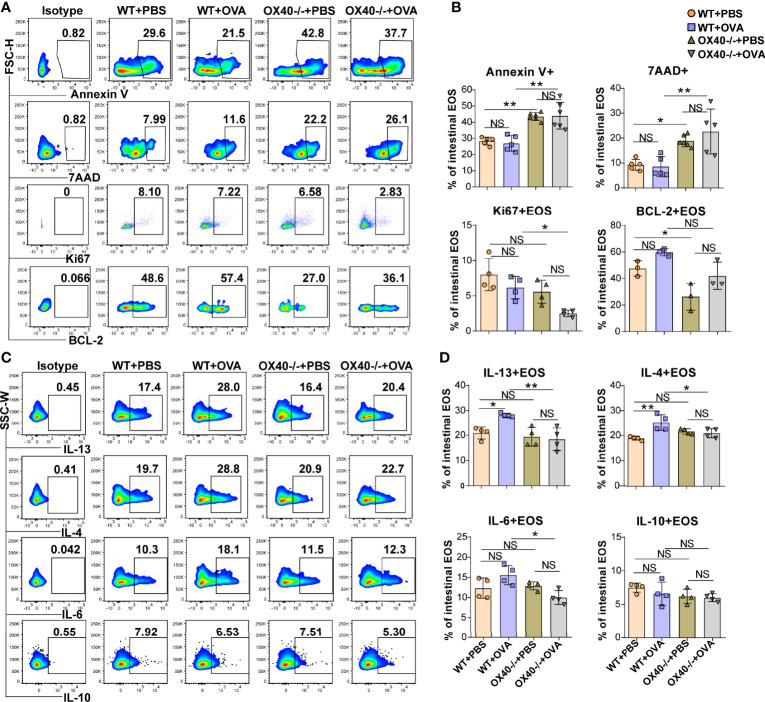
*Ox40* deficiency promotes apoptosis and inhibits the proliferation and cytokine production of gut eosinophils *in vivo*. Annexin V, 7AAD, Ki67, and BCL-2 of gut eosinophils in the four groups were analyzed by flow cytometry **(A)** and statistical analysis **(B)**. IL-13, IL-4, IL-6, and IL-10 expression in gut eosinophils were shown in the representative flow cytometry plot **(C)** and statistical analysis **(D)** of the four groups (n=3-5 mice/group). Experiments were repeated 2-3 times. Data are represented as the mean ± SD. *P < 0.05; **P < 0.01; NS, nonsignificant.

*Ox40* deficiency promoted apoptosis and reduced the proliferation of intestinal eosinophils. *Ox40* deficiency increased the proportion of apoptotic cells (Annexin V+) and dead cells (7AAD+) (**Figures 5A, B**) of intestinal eosinophils of PBS treated and OVA-induced EGE mice. Additionally, the expression of BCL-2 and Ki67 (**Figures 5A, B**) were decreased in *Ox40* ^–/–^ mice. The Ki67 expression in intestinal eosinophils of *Ox40^-/-^* +OVA mice was significantly lower than that of WT+OVA mice. A similar result of BCL-2 expression was observed that a lower level of BCL-2 in gut eosinophils was observed in *Ox40^-/-^* +PBS mice than the WT+PBS mice. And there was a lower trend of BCL-2 expression in gut eosinophils of *Ox40^-/-^* +OVA mice, compared to the WT+OVA mice. As for cytokine expression, intestinal eosinophils from *Ox40* ^–/–^ +OVA mice produced less IL-4, IL-6, and IL-13 (**Figures 5C, D**). While the IL-10 expression was similar among the four groups (**Figures 5C, D**). These data indicated that *Ox40* deficiency ameliorated OVA challenge-induced inflammation and reduced intestinal eosinophil infiltration, proliferation and inflammatory cytokine production.

### Ox40 Deficiency Impedes the Survival and Inflammatory Mediator Production of Eosinophils

**Figure 6:**
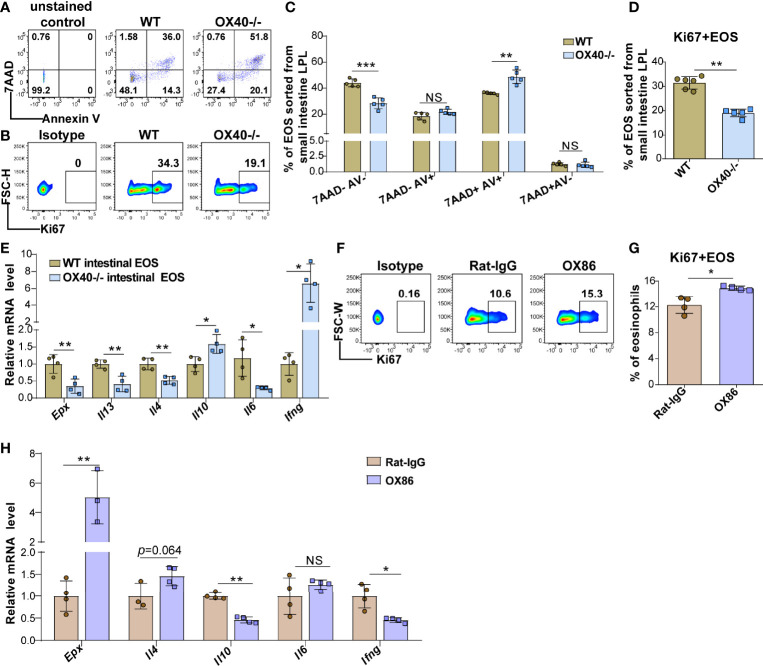
*Ox40* deficiency impedes the survival and inflammatory mediator production of gut eosinophils. Intestinal eosinophils of WT mice and *Ox40^-/-^* mice were sorted with a FACS Aria II cell sorter and stimulated with rmIL-5 or agonistic anti-OX40 antibody OX86. The Annexin V and 7AAD in gut eosinophils from WT mice and Ox40-/- mice were analyzed by flow cytometry **(A)** and statistical analysis **(B)** after being stimulated with IL-5 for 4 hours. **(C)** The representative flow cytometry scatter plot and statistical analysis **(D)** of Ki67 expression. **(E)** Relative mRNA levels of *Epx, Il4, Il13, Il10, Il6*, and *Ifng* in sorted gut eosinophils from WT mice and *Ox40^-/-^* mice. Sorted gut eosinophils of WT mice were stimulated with the agonistic anti-OX40 antibody OX86 or Rat-IgG for 6 hours (n=4-6 independent sample/group). Representative flow cytometric analysis **(F)** and statistical analysis **(G)** of Ki67 expression in the two groups. **(H)** Relative mRNA levels of *Epx, Il4, Il10, Il6*, and *Ifng* in Rat-IgG- and OX86-treated gut eosinophils (n=4-6 independent sample/group). Experiments were repeated twice. Data are represented as the mean ± SD. *P < 0.05; **P < 0.01; ***P < 0.001; NS, nonsignificant.

The above results showed that OVA challenge significantly upregulated OX40 expression in intestinal eosinophils and BM eosinophils and that *Ox40* knockout alleviated EGE inflammation. However, the effect of Th2 cells cannot be separated from systemic *Ox40* knockout. To further explore the impact of OX40 on eosinophils, intestinal eosinophils were sorted and unselected bone marrow progenitors separated from WT and *Ox40* ^-/-^ mice were cultured and stimulated with rmIL-5 *in vitro* to generate bone marrow-derived eosinophils (bmEos) for conducting the *in vitro* experiments. Sorted intestinal eosinophils of WT and *Ox40^-/-^* mice were stimulated with IL-5 for 4-6 hours to detect the expression of 7AAD, Annexin V, Ki67, and *Epx, Il4, Il13, Il6, Il10*, and *Ifng* mRNA levels. As shown in **Figures 6A, C**, the intestinal eosinophils of *Ox40^-/-^* mice have a higher proportion of late apoptosis cells (7AAD+Annexin V+), a lower proportion of live cells (7AAD-Annexin V-) and a lower level of Ki67 expression (**Figures 6B, D**). Besides, the mRNA levels of *Epx, Il4, Il13*, and *Il6* of sorted intestinal eosinophils of *Ox40^-/-^* mice were significantly lower than that of WT mice (**Figure 6E**).

The same phenomenon was observed in the bmEos. After 8-10 days of stimulation, compared to WT BM progenitor cells, *Ox40* ^-/-^ BM progenitor cells differentiated into fewer eosinophils (**Figures S7A, B** and **S8**). As shown in **Figures S7C, D**, compared to WT bmEos, *Ox40* ^-/-^ bmEos generated higher proportions of early apoptotic (7AAD-annexin V+) and late apoptotic (7AAD+annexin V+) bmEos, higher proportions of dead (7AAD+annexin V-) bmEos and lower proportion of live (7AAD-annexin V-) bmEos. Meanwhile, the proportion of Ki67-positive bmEos was decreased in *Ox40* ^-/-^ mice (**Figures S7E**). These data indicated that *Ox40* deficiency promoted eosinophil apoptosis and inhibited eosinophil proliferation and survival. Furthermore, compared to those from WT mice, rmIL-5-stimulated bmEos from *Ox40* ^-/-^ mice produced lower mRNA levels of *Epx, Il4, Il13*, and *Il6* and a higher mRNA level of *Il10* (**Figure S7F**). The results suggested that *Ox40* knockout attenuated the differentiation, proliferation, survival, and type 2 cytokine production of eosinophils.

### Oxc40 Agonist Ox86 Stimulation Facilitates Inflammatory Cytokine Production and Increases Eosinophil Survival *in Vitro*

To further explore the function of OX40 in eosinophils, naïve mature BM eosinophils and intestinal eosinophils from WT mice were sorted and stimulated with the agonistic anti-OX40 antibody OX86 for 6-12 hours. After stimulated with OX86, the intestinal eosinophils and BM eosinophils expressed a higher level of Ki67 (**Figures 6F-G** and **S7G**) than Rat-IgG isotype-treated eosinophils. Moreover, OX86 stimulation significantly increased *Epx, Il4, Il12p35*, and *Il6* mRNA levels and decreased *Il10* and *Ifng* mRNA levels in BM eosinophils (**Figure S7H**). Similarly, OX86 stimulated intestinal eosinophils exhibited higher level of *Epx* mRNA and an increased trend of *Il4* mRNA level, and lower levels of *Il10* and *Ifng* mRNA (**Figure 6H**). These data indicate that the expression or activation of OX40 promotes the survival of eosinophils, facilitates the type 2 inflammatory reaction of eosinophils, and mitigates the Th1 inflammatory reaction.

### OX40 Promotes Eosinophil-Induced Inflammation *via* the Traf2/6-Related NF-κB/RelA Pathway.

**Figure 7:**
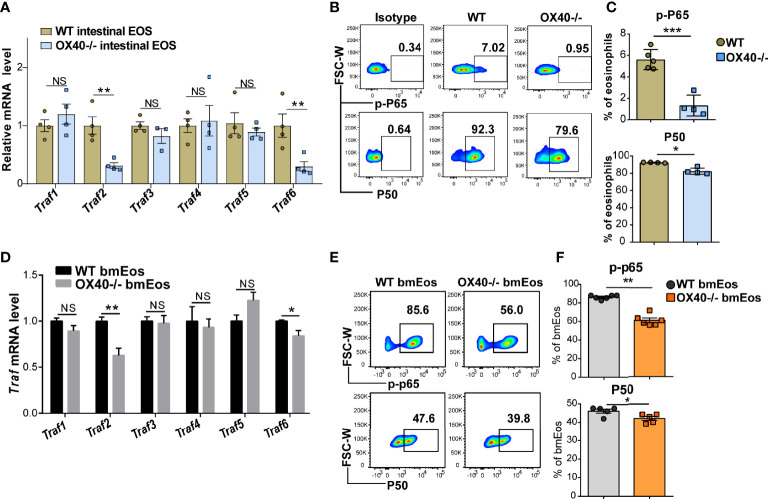
OX40 promotes eosinophil-induced inflammation *via* the Traf2/6-related NF-κB/RelA pathway. Gut eosinophils from C57BL/6J WT and *Ox40^-/-^* mice were sorted with a FACS Aria II cell sorter and stimulated with 30 ng/ml IL-5 for one or six hours. Cultured bmEos from C57BL/6J WT and *Ox40^-/-^* mice were sorted with a FACS Aria II cell sorter. **(A)** Relative mRNA levels of *Traf1, Traf2, Traf3, Traf4, Traf5*, and *Traf6* in WT and *Ox40^-/-^* gut eosinophils by stimulated with IL-5 for 6 hours. Flow cytometric analysis **(B)** and statistical analysis **(C)** of phosphorylated RelA (P65) and the P50 protein. **(D)** Relative mRNA levels of *Traf1, Traf2, Traf3, Traf4, Traf5*, and *Traf6* in WT and *Ox40^-/-^* bmEos on Day 9. Flow cytometric analysis **(E)** and statistical analysis **(F)** of phosphorylated RelA (P65) and the P50 protein. Experiments were repeated twice. Data are represented as the mean ± SEM. *P < 0.05; **P < 0.01; ***P < 0.001; NS, nonsignificant.

The OX40-OX40L interaction activates Trafs family members and downstream NF-κB signaling pathways in T cells (11, 25) and neutrophils (15). To clarify whether the Trafs family and downstream NF-κB signaling pathways are associated with the role of OX40 in eosinophils, the gene expression of Traf1/2/3/4/5/6 was detected in sorted intestinal eosinophils and cultured bmEos from WT and *Ox40* ^–/–^ mice. After rmIL-5 stimulation for 6 hours, the Traf2 and Traf6 mRNA expression was significantly decreased in intestinal eosinophils of *Ox40* ^–/–^ mice (**Figure 7A**), as well as the phosphorylation of RelA (P65) and protein P50 (**Figures 7B, C**). The same results were observed in bmEos of WT and *Ox40* ^–/–^ mice. Stimulated with IL-5 for 5 days (On Day 9), the Traf2/6 mRNA expression was significantly decreased in *Ox40* ^–/–^ bmEos compared to WT bmEos (**Figure 7D**). Furthermore, *Ox40* knockout significantly decreased the phosphorylation of RelA (P65) and the protein expression of P50 (**Figures 7E, F**) of bmEos. Thus, OX40 regulated the function of eosinophils by inhibiting the Traf2/6-associated NF-κB pathway.

## Discussion

Allergy sensitization triggers food allergen-specific IgE and Th2 cells, together with eosinophils, basophils, mast cells and antigen-presenting cells, which contribute to the development of EGIDs (4). Eosinophils have multiple functions in health and disease (26). Eosinophils act as antigen-presenting cells to regulate Th1 and Th2 immune responses (27, 28) and promote gastrointestinal inflammation by synthesizing, storing and releasing cytokines, chemokines and lipid mediators (10, 29). However, excessive accumulation of eosinophils in the gastrointestinal tract leads to tissue damage *via* the release of toxic granule proteins, such as MBP and EPX, and inflammatory cytokines (10). No therapy for the management of EGIDs is currently approved by the U.S. Food and Drug Administration (FDA). Major treatment strategies for EGIDs, including diet therapy, steroid therapy, anti-IL-5 treatment, and anti-IL-13 treatment, lack efficacy, indicating that the pathogenesis of EGIDs requires further exploration (30).

The interaction between OX40 expressed by T cells (31) and OX40L expressed by antigen-presenting cells or airway smooth muscle cells (32) plays a key role in the pathogenesis of allergic diseases, such as asthma (33) and atopic dermatitis (34). The OX40/OX40L pathway leads to the development of pathogenic Th2 cells in mouse asthma (35). Similar phenomena were observed in OX40-OX40L blockade-treated gastrointestinal nematode infection (36). Consistent with previous works, our study showed that *Ox40* deficiency significantly alleviated OVA-induced EGE. The common mechanism among asthma, parasitic infection and EGIDs is the domination by Th2 cells, with eosinophils as the main inflammatory effector cells. However, the relationship between OX40 and eosinophils is unclear. Interestingly, we observed dramatically increased OX40 expression in EGE eosinophils, even though OX40 was rarely expressed in normal eosinophils. Similarly, sorted mature BM eosinophils stimulated with rmIL-5 exhibited up-regulated OX40 expression. These results suggest that OX40 is expressed in not only T cells, macrophages and neutrophils but also eosinophils and that it may play a critical role in eosinophils in EGE mice.

OX40 expressed on T cells and neutrophils participates in adaptive immunity and innate immunity by regulating the activation, differentiation, cytokine production, and survival of cells (11, 13, 15). Further exploration is required to identify the function of OX40 in eosinophils. OVA challenge induced intestinal eosinophils and BM eosinophils to express high levels of OX40 and synthesize more type 2 cytokines. Furthermore, *Ox40* deficiency significantly reduced OVA-induced eosinophil infiltration and inflammatory factor and BCL-2 expression in eosinophils *in vivo*. These results further indicate that OX40 is related to tissue eosinophil infiltration and function in the EGE model. To further clarify the role of OX40 in the function of eosinophils, a series of experiments were performed *in vitro*. The OX40-OX40L interaction can induce a Th1 or Th2 immune response in different diseases, depending on the environment (37). IL-5 is the most specific cytokine that promotes the differentiation, proliferation, survival and activation of eosinophils (5, 22, 38) and affects the migration, activation and function of eosinophils in inflamed tissues (26). To simulate the type 2 reaction environment of EGE, intestinal eosinophils were sorted from WT and *Ox40^-/-^* mice and stimulated with rmIL-5. And the bone marrow precursor cells were extracted from WT and *Ox40^-/-^* mice and cultured *in vitro* with IL-5 stimulation. After activation by IL-5, the BM cells of *Ox40^-/-^* mice exhibited less differentiation into eosinophils. Moreover, the intestinal eosinophils and bmEos of *Ox40^-/-^* mice showed increased apoptosis and reduced proliferation and less pro-inflammatory cytokines production, including *Epx, Il4, Il13*, and *Il6*, and more protective factors *Il10* and Th1 cytokine *Ifng* production. All these conversions were reversed in sorted WT intestinal eosinophils and sorted bone marrow eosinophils by the agonistic anti-OX40 antibody OX86 stimulation. These data indicate that the OX40 pathway reduces eosinophil apoptosis, promotes eosinophil proliferation and survival, and enhances the type 2 immune response mediated by eosinophils. These results revealed a new process of eosinophil activation in which eosinophils can be activated by other cells expressing OX40L through the OX40-OX40L interaction in EGE. Thus, an OX40-related molecule targeting eosinophils may be a promising agent for EGIDs treatment in the future.

Previous studies have shown that the NF-κB signaling pathway promotes the survival of eosinophils by regulating the BCL-XL (39) and BCL-2 (40) families. Moreover, OX40 regulates the apoptosis, proliferation, differentiation and inflammatory factor production of T cells and neutrophils through the Traf family-related NF-κB signaling pathway (11, 15). As our data showed, *Ox40* deficiency decreased the mRNA expression of Traf2/6 in sorted intestinal eosinophils and cultured bmEos. In addition, downstream NF-κB signaling pathway-related molecules were also significantly reduced in *Ox40* ^-/-^ bmEos and intestinal eosinophils, including phosphorylated RelA (P65) and the P50 protein. All these data suggest that the Traf2/6-related NF-κB signaling pathway is downstream of OX40 and may play a central role in the OX40-related proliferation, apoptosis and cytokine production of eosinophils.

However, our research had some limitations. OX40 was deleted in T cells, neutrophils, macrophages, eosinophils and other cells in *Ox40^-/-^* mice; thus, the effect of *Ox40* knockout on OVA-challenged mice may reflect synergy among several immune cell types. An *Ox40* conditional knockout mouse is worthy of further study.

## Conclusions

Our recent work revealed a significantly upregulated OX40 expression in eosinophils of mouse EGE. Moreover, a new mechanism by which OX40 regulates eosinophils has been discovered: OX40 aggravates eosinophil-mediated inflammatory damage by reducing apoptosis and increasing proliferation and type 2 cytokine production in eosinophils *via* the Traf2/6-related NF-κB signaling pathway. These findings provide new potential therapeutic targets for eosinophilic gastroenteritis.

## Data Availability Statement

The original contributions presented in the study are included in the article/**Supplementary Material**. Further inquiries can be directed to the corresponding authors.

## Ethics Statement

The animal study was reviewed and approved by Institutional Animal Care and Ethics Committee at Peking University Ninth Clinical College and Beijing Friendship Hospital.

## Author Contributions

All listed authors participated meaningfully in the study and that they have seen and approved the submission of this manuscript. LX and DT participated in performing the research, analyzing the data, and initiating the original draft of the article. MZ, JM, GS, H.J and ML participated in performing the research. JW, and DZ established the hypotheses, supervised the studies and reviewed the manuscript. All authors contributed to the article and approved the submitted version.

## Funding

This work was supported by grants from the Youth Beijing Scholar (No. 035), the Beijing Municipal Administration of Hospitals’ Youth Program (No. QMS20200103), the Scientific Research Common Program of Beijing Municipal Commission of Education (No. KM202110025015), the Digestive Medical Coordinated Development Center of Beijing Hospitals Authority (No. XXZ015), the Science and Technology Development Project of China State Railway Group (No. N2019Z004), the Special Scientific Research Fund for Tutor of Beijing Friendship Hospital (No. YYDSZX201901), and the Beijing Science and Technology Program (No. Z211100002921028).

## Conflict of Interest

The authors declare that the research was conducted in the absence of any commercial or financial relationships that could be construed as a potential conflict of interest.

## Publisher’s Note

All claims expressed in this article are solely those of the authors and do not necessarily represent those of their affiliated organizations, or those of the publisher, the editors and the reviewers. Any product that may be evaluated in this article, or claim that may be made by its manufacturer, is not guaranteed or endorsed by the publisher.

## Supplementary Material

Supplementary Figure 1 The cell surface proteins expression in spleen cells treated with collagenase or lysing solution. Half spleen cells were treated with lysing solution to lyse the red blood cells for 3 minutes, then the cells were treated with collagenase D (collagenase group) or PBS solution (control group) for twenty minutes. The rest of the spleen cells were treated with PBS solution, and then we detected the cell surface proteins, including F4/80, Ly6G, Siglec-F, CD45, OX40, and CD11b.

Supplementary Figure 2 The H&E staining of the intestine tissues. **(A)** The H&E staining of the intestine tissues of mice in the PBS group and the OVA group. The blue arrows point to the inflammatory site. **(B)** The H&E staining of the intestine tissues of mice in the WT+PBS group, the WT+OVA group, the *Ox40^-/-^* +PBS group, and the *Ox40^-/-^* +OVA group.

Supplementary Figure 3 Gating strategy of eosinophils in gut tissue and spleen and mLNs. **(A)** The gating strategy of eosinophils in the small intestine LPL and colon LPL. **(B)** The gating strategy of eosinophils in spleen and mLNs.

Supplementary Figure 4 Eosinophils infiltration in mLNs, spleen and peripheral blood. Representative flow cytometry scatter plot **(A)** and statistical analysis **(B)** of eosinophils infiltrated in mLNs, spleen and peripheral blood among the three groups (n=5 mice/group). Experiments were repeated 2-3 times. Data are represented as the mean ± SD. *P < 0.05; **P < 0.01; NS, nonsignificant.

Supplementary Figure 5 OX40 expression in sorted mature bone marrow eosinophils was increased after being stimulated with IL-5. The mature bone marrow eosinophils were sorted with a FACS Aria II cell sorter, gating at 7AAD- FceRIa-Siglec-F+CD11bint cells, and stimulated with IL-5 for 12-24 hours. Flow cytometric analysis **(A)** and statistical analysis **(B)** of the median fluorescence intensities (MFI) of OX40. Experiments were repeated twice. Data are represented as the mean ± SD. *P < 0.05; **P < 0.01.

Supplementary Figure 6 The cytokine production of mLNs eosinophils. Representative flow cytometry scatter plot **(A)** and statistical analysis **(B)** of IL-13, IL-4 and TNF-a expression in mLNs eosinophils of the control and OVA challenged group (n=4-5 mice/group). Experiments were repeated2-3 times. Data are represented as the mean ± SD. *P < 0.05; **P < 0.01; NS, nonsignificant.

Supplementary Figure 7
*Ox40* deficiency impedes the survival and inflammatory mediator production of bmEos *in vitro*. Unselected bone marrow progenitors from WT mice and *Ox40* ^-/-^ mice were cultured and stimulated with rmIL-5 *in vitro* to generate bmEos. The generation of 7AAD-SiglecF+SSA-C^high^ bmEos from WT mice and *Ox40* ^-/-^ mice was detected by flow cytometry and the representative flow cytometry plot of bmEos on Day 10 was shown in **(A)**. **(B)** Statistical analysis of the percentage and number of generated bmEos from WT mice and *Ox40* ^-/-^ mice at different time points (n=4 mice/group). On Day 10, live bmEos (7AAD-SiglecF+SSA-C^high^) were sorted with a FACS Aria II cell sorter (purity > 97%) for the following research. Annexin V and 7AAD in bmEos from WT mice and *Ox40* ^-/-^ mice was analyzed by flow cytometry **(C)** and statistical analysis **(D)** on Day 10. **(E)** The representative flow cytometry scatter plot and statistical analysis of Ki67 expression in bmEos from WT mice and *Ox40* ^-/-^ mice on Day 10. **(F)** Relative mRNA levels of *Epx, Il4, Il13, Il10, Il6*, and *Ifng* in sorted bmEos from WT mice and *Ox40* ^-/-^ mice (n=4-6 independent sample/group). Naïve mature BM eosinophils sorted from BM cells of C56BL/6J WT mice (purity > 90%) were stimulated with the agonistic anti-OX40 antibody OX86 or Rat-IgG for 12 hours (n=4-6 independent sample/group). **(G)** Representative flow cytometric analysis and statistical analysis of Ki67 expression in the two groups. **(H)** Relative mRNA levels of *Epx, Il4, Il10, Il12p35, Il6*, and *Ifng* in Rat-IgG- and OX86-treated BM eosinophils. Experiments were repeated 2-3 times. Data are represented as the mean ± SD. *P < 0.05; **P < 0.01; NS, nonsignificant.

Supplementary Figure 8 OX40 deficiency generates fewer bmEos induced by IL-5 *in vitro*. To explore the effect of OX40 on IL-5-induced bmEos generation *in vitro* and eliminate the impact of the initial number of eosinophils, the total cell count and the relative cell count and the percentage of bmEos at different time points were analyzed. The relative percentage **(A)** and cell count **(B)** of bmEos (fold change relative to Day 0) from WT and Ox40-/- mice (n=4 mice/group). **(C)** The total count of the bone marrow cells of WT and OX40-/- mice. Experiments were repeated 2-3 times. Data are represented as the mean ± SD. *P < 0.05; **P < 0.01; NS, nonsignificant.

## Abbreviations

BM, bone marrow; bmEos, bone marrow-derived eosinophils; *Ccr3*, chemokine (C-C motif) receptor 3;*Ccl11*, chemokine (C-C motif) ligand 11;EGIDs, eosinophilic gastrointestinal diseases; EOS, eosinophils; EPX, eosinophil peroxidase; ECP, eosinophil cationic protein; EDN, eosinophil-derived neurotoxin; EGE, eosinophilic gastroenteritis; ELISA, enzyme-linked immunosorbent assay; H&E, hematoxylin-eosin; IL-4, interleukin-4; IL-13, interleukin-13; IL-5, interleukin-5; IL-6, interleukin-6; IL-10, interleukin-10; Ifng, Interferon-gama; LPL, lamina propria layer; MLNs, mesenteric lymph nodes; MBP, major basic protein; MFI, median fluorescence intensities; NF- κB, nuclear factor kappa-B; OVA, ovalbumin; p-p65, phosphorylation of RelA; P50, the protein P50; Trafs, TNFR-associated factors; TSLP, thymopoietin; TNF-α, tumor necrosis factor-alpha.
