# Supplementary figures and images for: OX40 Expression in Eosinophils Aggravates OVA-Induced Eosinophilic Gastroenteritis
Source: Front Immunol. 2022 Jun 2;13:841141. doi: 10.3389/fimmu.2022.841141 (PMC9201343; doi:10.3389/fimmu.2022.841141)

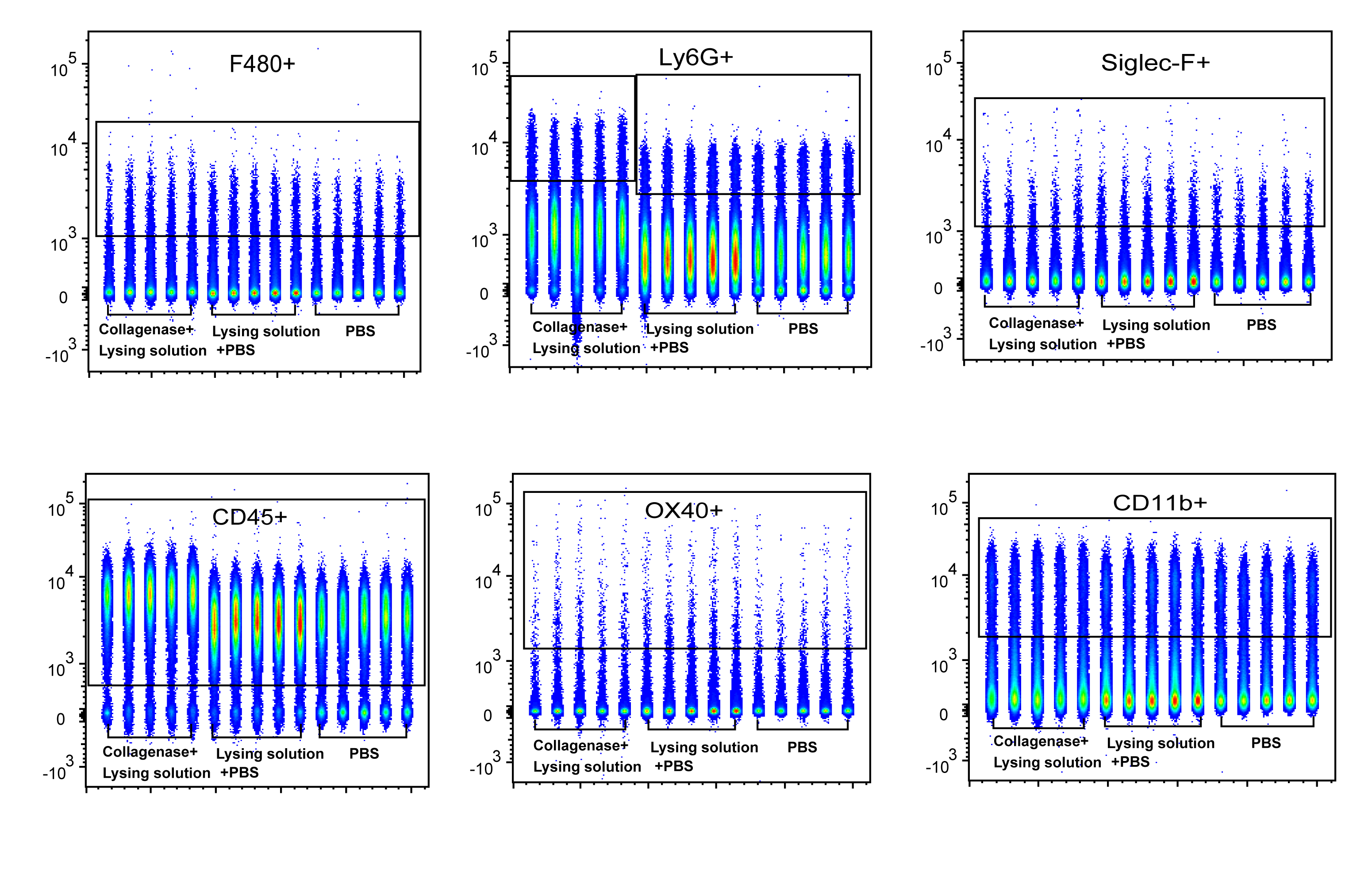

Supplement: Supplementary Figure 1 — The cell surface proteins expression in spleen cells treated with collagenase or lysing solution. Half spleen cells were treated with lysing solution to lyse the red blood cells for 3 minutes, then the cells were treated with collagenase D (collagenase group) or PBS solution (control group) for twenty minutes. The rest of the spleen cells were treated with PBS solution, and then we detected the cell surface proteins, including F4/80, Ly6G, Siglec-F, CD45, OX40, and CD11b. [file Image_1.tif]

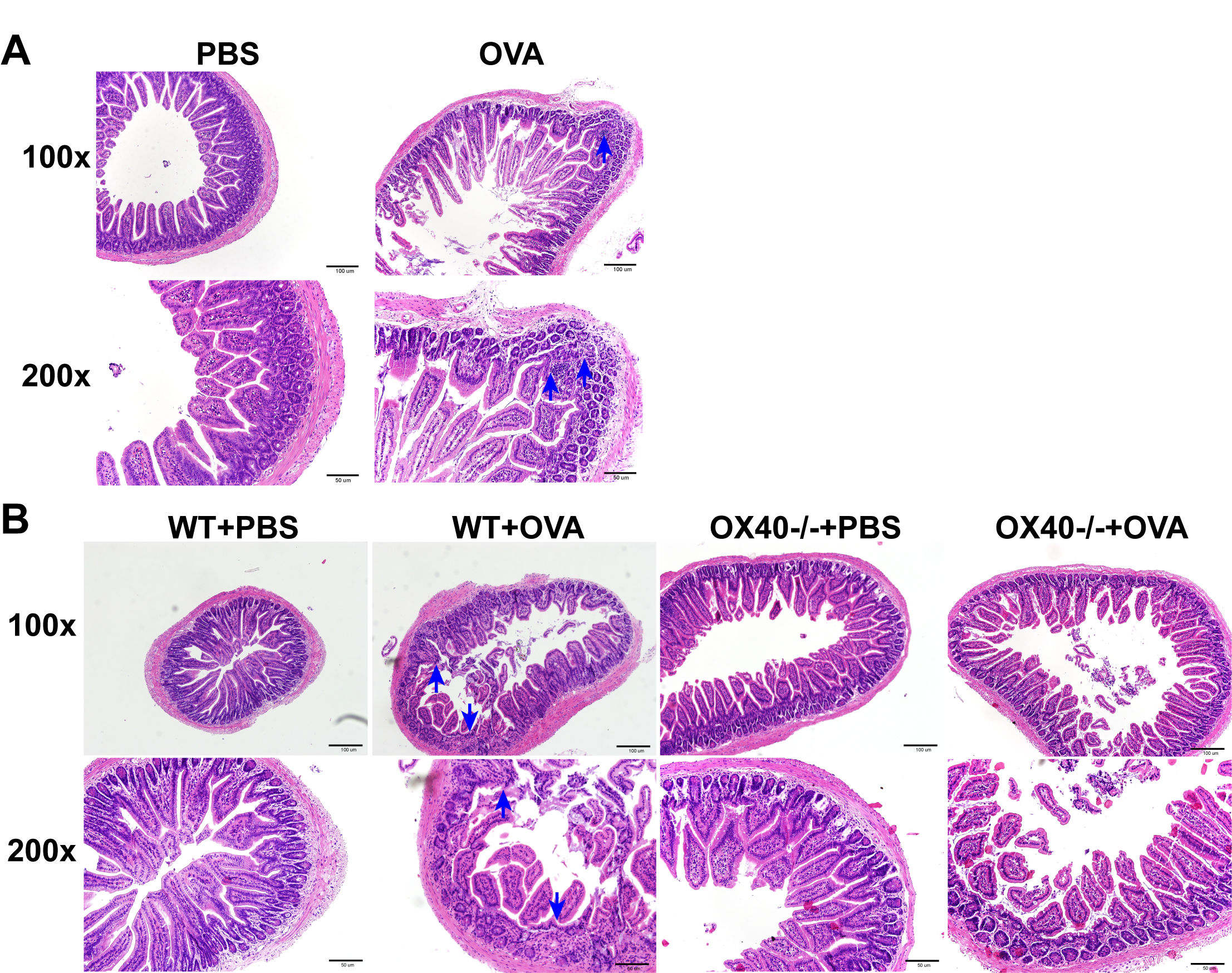

Supplement: Supplementary Figure 2 — The H&E staining of the intestine tissues. (A) The H&E staining of the intestine tissues of mice in the PBS group and the OVA group. The blue arrows point to the inflammatory site. (B) The H&E staining of the intestine tissues of mice in the WT+PBS group, the WT+OVA group, the Ox40-/- +PBS group, and the Ox40-/- +OVA group. [file Image_2.tif]

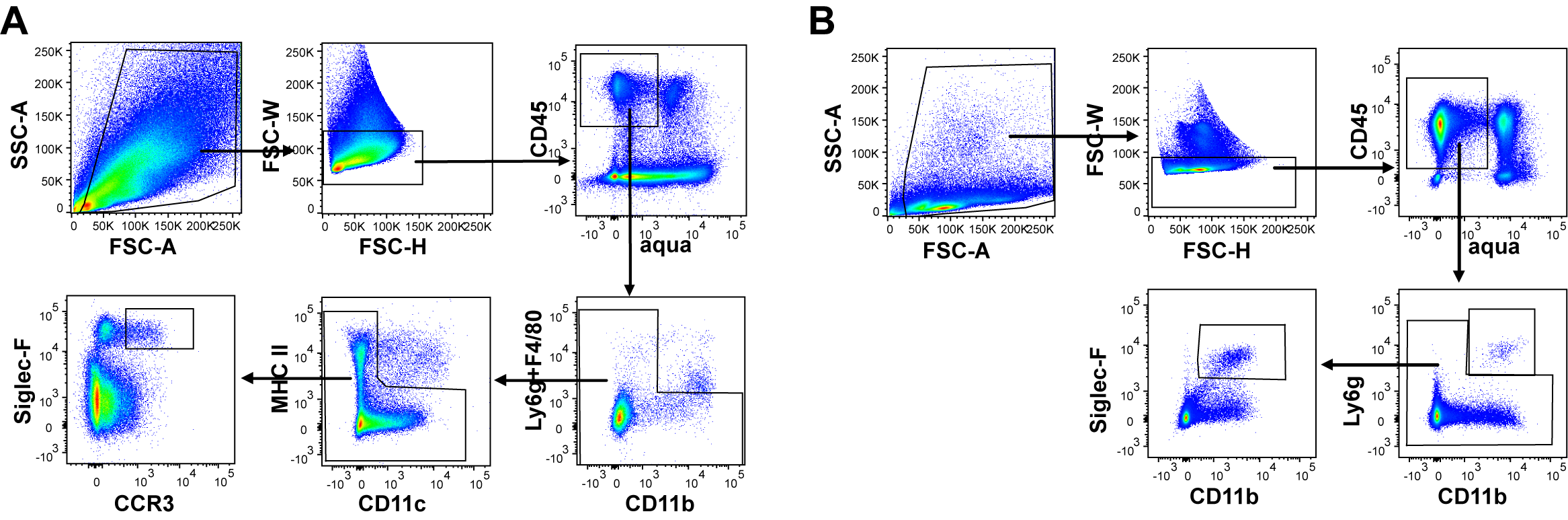

Supplement: Supplementary Figure 3 — Gating strategy of eosinophils in gut tissue and spleen and mLNs. (A) The gating strategy of eosinophils in the small intestine LPL and colon LPL. (B) The gating strategy of eosinophils in spleen and mLNs. [file Image_3.tif]

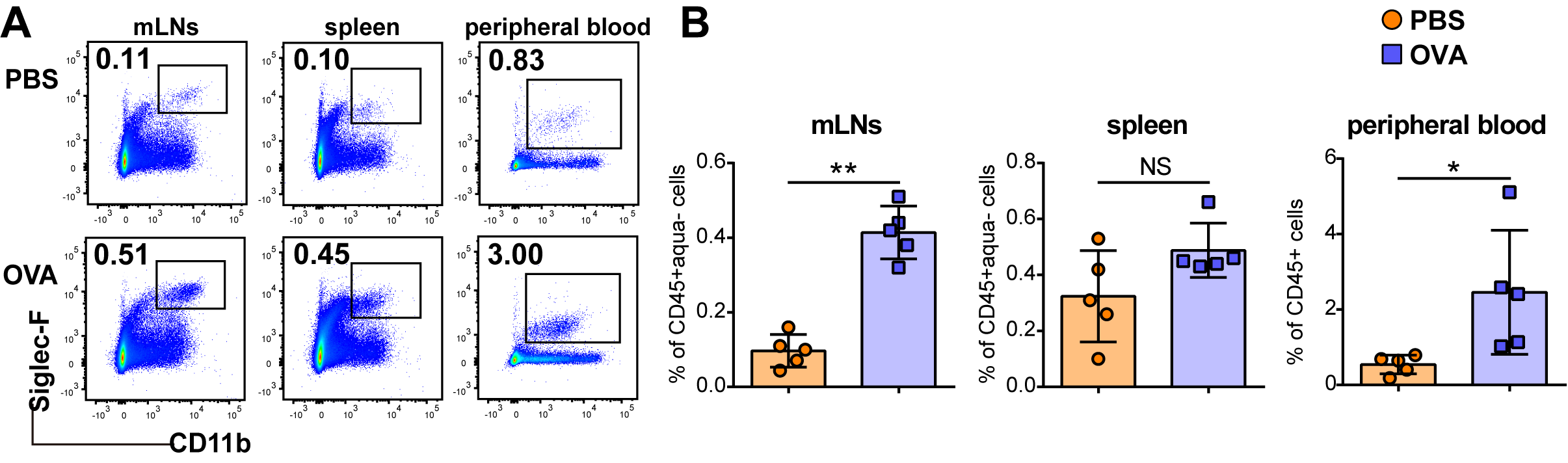

Supplement: Supplementary Figure 4 — Eosinophils infiltration in mLNs, spleen and peripheral blood. Representative flow cytometry scatter plot (A) and statistical analysis (B) of eosinophils infiltrated in mLNs, spleen and peripheral blood among the three groups (n=5 mice/group). Experiments were repeated 2-3 times. Data are represented as the mean ± SD. *P < 0.05; **P < 0.01; NS, nonsignificant. [file Image_4.tif]

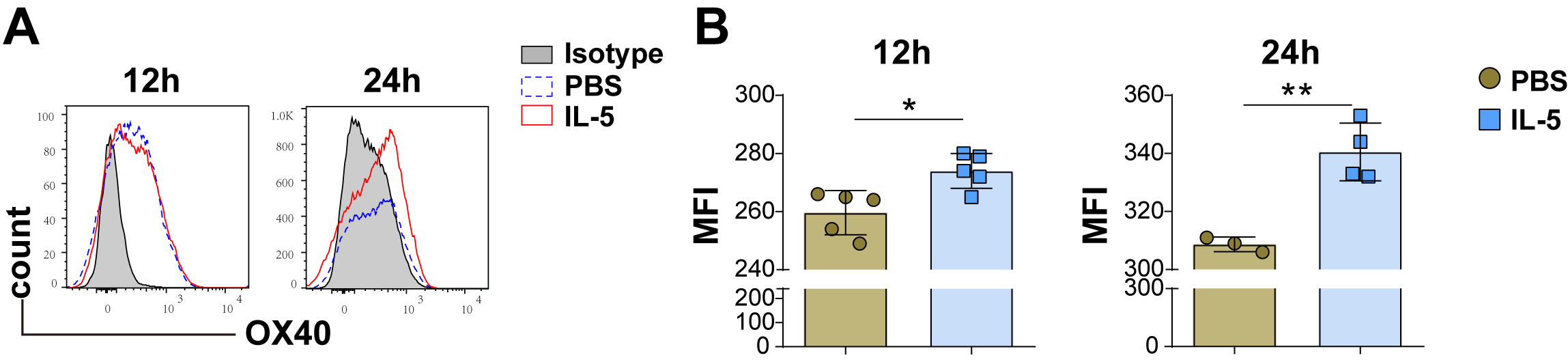

Supplement: Supplementary Figure 5 — OX40 expression in sorted mature bone marrow eosinophils was increased after being stimulated with IL-5. The mature bone marrow eosinophils were sorted with a FACS Aria II cell sorter, gating at 7AAD- FceRIa-Siglec-F+CD11bint cells, and stimulated with IL-5 for 12-24 hours. Flow cytometric analysis (A) and statistical analysis (B) of the median fluorescence intensities (MFI) of OX40. Experiments were repeated twice. Data are represented as the mean ± SD. *P < 0.05; **P < 0.01. [file Image_5.tif]

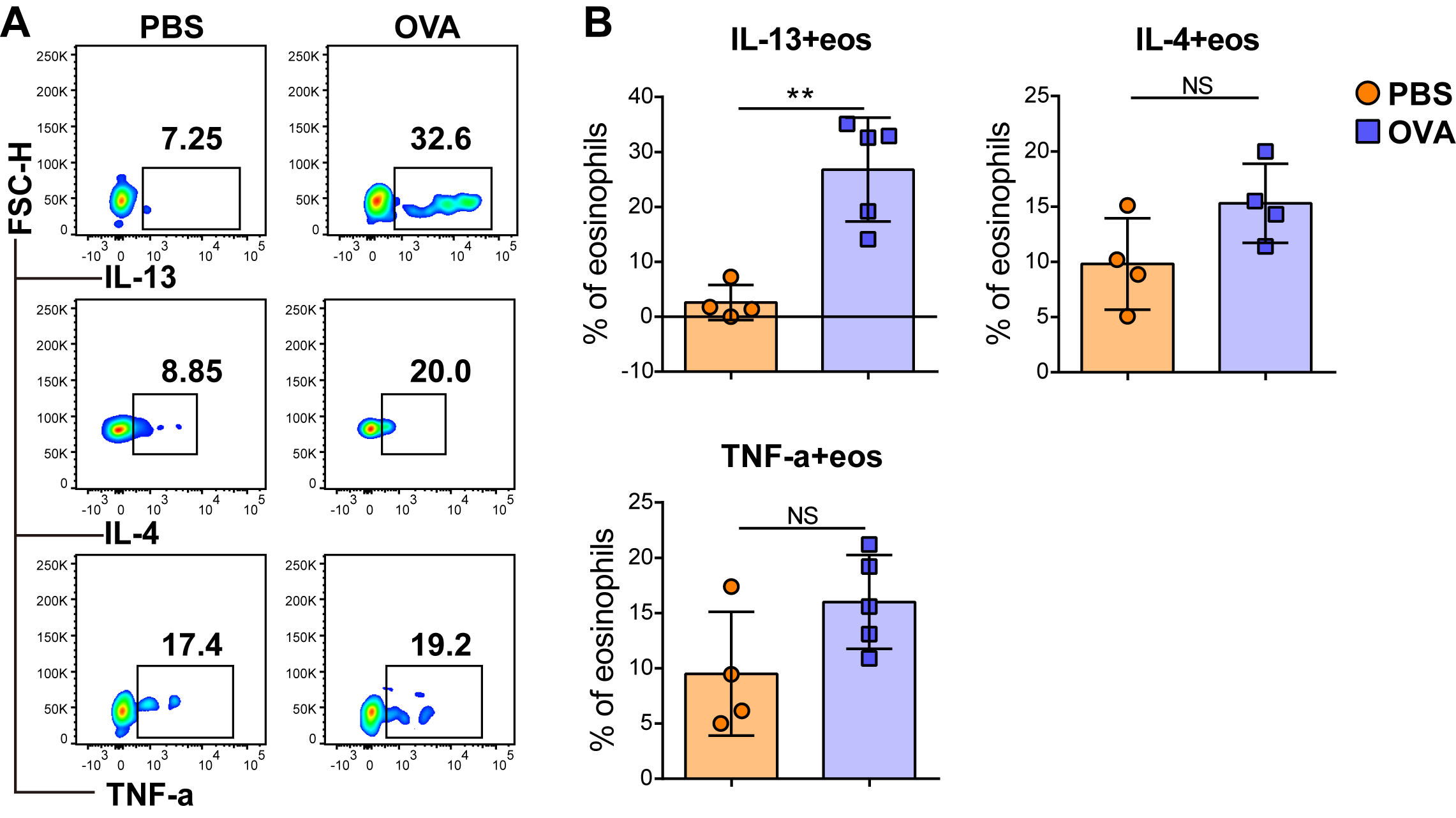

Supplement: Supplementary Figure 6 — The cytokine production of mLNs eosinophils. Representative flow cytometry scatter plot (A) and statistical analysis (B) of IL-13, IL-4 and TNF-a expression in mLNs eosinophils of the control and OVA challenged group (n=4-5 mice/group). Experiments were repeated2-3 times. Data are represented as the mean ± SD. *P < 0.05; **P < 0.01; NS, nonsignificant. [file Image_6.tif]

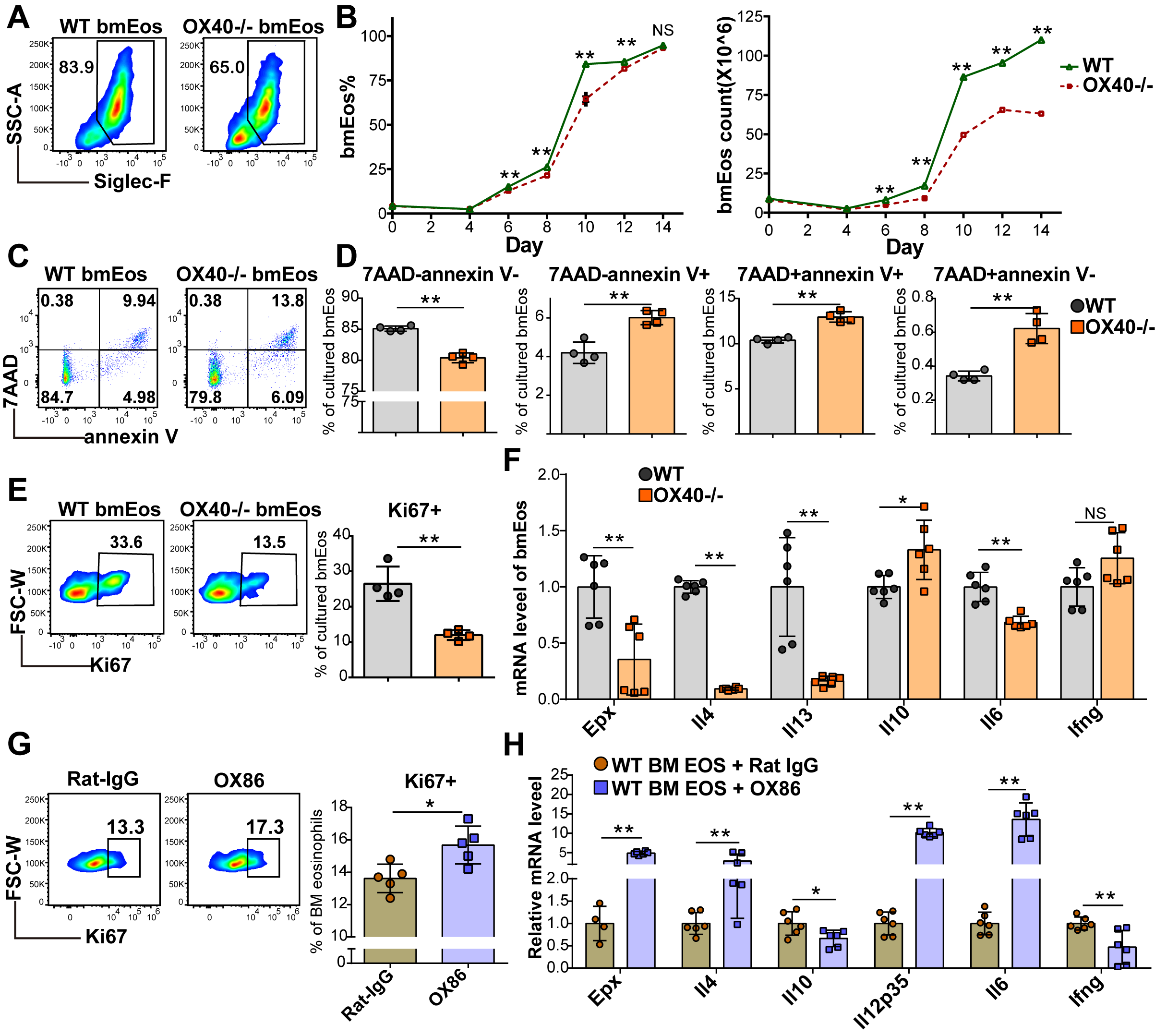

Supplement: Supplementary Figure 7 — Ox40 deficiency impedes the survival and inflammatory mediator production of bmEos in vitro. Unselected bone marrow progenitors from WT mice and Ox40 -/- mice were cultured and stimulated with rmIL-5 in vitro to generate bmEos. The generation of 7AAD-SiglecF+SSA-Chigh bmEos from WT mice and Ox40 -/- mice was detected by flow cytometry and the representative flow cytometry plot of bmEos on Day 10 was shown in (A). (B) Statistical analysis of the percentage and number of generated bmEos from WT mice and Ox40 -/- mice at different time points (n=4 mice/group). On Day 10, live bmEos (7AAD-SiglecF+SSA-Chigh) were sorted with a FACS Aria II cell sorter (purity > 97%) for the following research. Annexin V and 7AAD in bmEos from WT mice and Ox40 -/- mice was analyzed by flow cytometry (C) and statistical analysis (D) on Day 10. (E) The representative flow cytometry scatter plot and statistical analysis of Ki67 expression in bmEos from WT mice and Ox40 -/- mice on Day 10. (F) Relative mRNA levels of Epx, Il4, Il13, Il10, Il6, and Ifng in sorted bmEos from WT mice and Ox40 -/- mice (n=4-6 independent sample/group). Naïve mature BM eosinophils sorted from BM cells of C56BL/6J WT mice (purity > 90%) were stimulated with the agonistic anti-OX40 antibody OX86 or Rat-IgG for 12 hours (n=4-6 independent sample/group). (G) Representative flow cytometric analysis and statistical analysis of Ki67 expression in the two groups. (H) Relative mRNA levels of Epx, Il4, Il10, Il12p35, Il6, and Ifng in Rat-IgG- and OX86-treated BM eosinophils. Experiments were repeated 2-3 times. Data are represented as the mean ± SD. *P < 0.05; **P < 0.01; NS, nonsignificant. [file Image_7.tif]

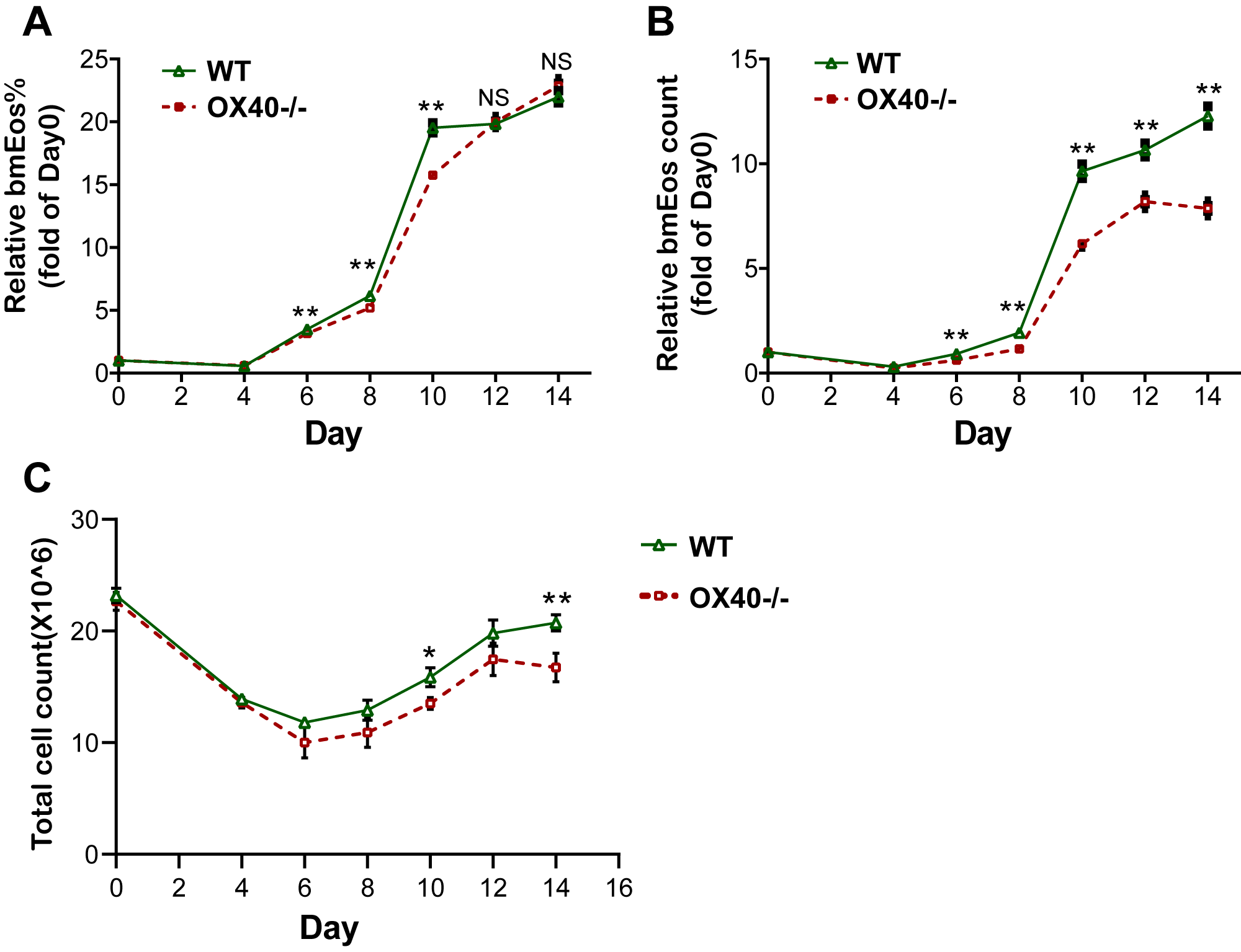

Supplement: Supplementary Figure 8 — OX40 deficiency generates fewer bmEos induced by IL-5 in vitro. To explore the effect of OX40 on IL-5-induced bmEos generation in vitro and eliminate the impact of the initial number of eosinophils, the total cell count and the relative cell count and the percentage of bmEos at different time points were analyzed. The relative percentage (A) and cell count (B) of bmEos (fold change relative to Day 0) from WT and Ox40-/- mice (n=4 mice/group). (C) The total count of the bone marrow cells of WT and OX40-/- mice. Experiments were repeated 2-3 times. Data are represented as the mean ± SD. *P < 0.05; **P < 0.01; NS, nonsignificant. [file Image_8.tif]
